# Dysregulation of RNA-Binding Proteins in Amyotrophic Lateral Sclerosis

**DOI:** 10.3389/fnmol.2020.00078

**Published:** 2020-05-29

**Authors:** Yuan Chao Xue, Chen Seng Ng, Pinhao Xiang, Huitao Liu, Kevin Zhang, Yasir Mohamud, Honglin Luo

**Affiliations:** ^1^Centre for Heart and Lung Innovation, St. Paul’s Hospital, Vancouver, BC, Canada; ^2^Department of Pathology and Laboratory Medicine, University of British Columbia, Vancouver, BC, Canada; ^3^Department of Experimental Medicine, University of British Columbia, Vancouver, BC, Canada

**Keywords:** aggregation, gene mutations, nucleocytoplasmic transport, RNA-binding proteins, RNA metabolism

## Abstract

Genetic analyses of patients with amyotrophic lateral sclerosis (ALS) have revealed a strong association between mutations in genes encoding many RNA-binding proteins (RBPs), including *TARDBP*, *FUS*, *hnRNPA1*, *hnRNPA2B1*, *MATR3*, *ATXN2*, *TAF15*, *TIA-1*, and *EWSR1*, and disease onset/progression. RBPs are a group of evolutionally conserved proteins that participate in multiple steps of RNA metabolism, including splicing, polyadenylation, mRNA stability, localization, and translation. Dysregulation of RBPs, as a consequence of gene mutations, impaired nucleocytoplasmic trafficking, posttranslational modification (PTM), aggregation, and sequestration by abnormal RNA foci, has been shown to be involved in neurodegeneration and the development of ALS. While the exact mechanism by which dysregulated RBPs contribute to ALS remains elusive, emerging evidence supports the notion that both a loss of function and/or a gain of toxic function of these ALS-linked RBPs play a significant role in disease pathogenesis through facilitating abnormal protein interaction, causing aberrant RNA metabolism, and by disturbing ribonucleoprotein granule dynamics and phase transition. In this review article, we summarize the current knowledge on the molecular mechanism by which RBPs are dysregulated and the influence of defective RBPs on cellular homeostasis during the development of ALS. The strategies of ongoing clinical trials targeting RBPs and/or relevant processes are also discussed in the present review.

## Introduction

Amyotrophic lateral sclerosis (ALS), a devastating neurodegenerative disease that primarily targets motor neurons, is categorized into two forms, familial (genetically inherited) and sporadic (without apparent family history). Familiar ALS (fALS) is responsible for ~5% to 10% of all ALS cases, whereas sporadic ALS (sALS) is the major form of the disease accounting for 90% to 95% of all cases (Brown and Al-Chalabi, 2017; van Es et al., 2017). Since the first identification of *SOD1* as a causative gene for ALS, the list of genetic mutations associated with ALS has grown rapidly. Up to date, more than 30 genes have been recognized as potential causal drivers for ALS (Al-Chalabi et al., 2017). Among them, many encode RNA-binding proteins (RBPs), such as transactivation response DNA-binding protein 43 (TDP-43) and fused in sarcoma/translocated in sarcoma (FUS/TLS).

As key regulators of RNA metabolism, RBPs play a vital role in maintaining the normal function of neuronal systems (Nussbacher et al., 2019). Under physiological conditions, these ALS-linked RBPs are involved in almost all aspects of RNA metabolism, including transcription, alternate splicing, mRNA transport, and stability. Although the exact functions and mechanisms of action of these RBPs are still largely unclear, current evidence suggests a central role for RBPs in the maintenance of neuronal integrity. Defects in RBPs have emerged as a significant contributing factor to the pathogenesis of ALS (Nussbacher et al., 2019). More notably, cytoplasmic mislocalization, aggregation, and fragmentation of RBPs, in particular TDP-43 (termed TDP-43 proteinopathies), have been regarded as a pathological hallmark of ALS or frontotemporal dementia (FTD, a disease sharing many genetic and pathological features with ALS; Neumann et al., 2006; Mackenzie et al., 2010). In this review article, we summarize the current understanding of how RBPs are dysregulated and the role of disrupted RBPs in ALS development. We also highlight the emerging therapeutic intervention by targeting these ALS-implicated RBPs.

## Mechanisms Leading to Dysregulation of RBPs in ALS

As alluded to above, mutations in genes encoding many RBPs are highly associated with ALS. In addition, dysregulation of RBPs as a result of compromised nucleocytoplasmic trafficking, posttranslational modification (PTM), aggregation, and sequestration by abnormal RNAs also contributes significantly to disease pathogenesis. This section will briefly discuss these underlying mechanisms resulting in RBP dysregulation in ALS.

### Gene Mutations

**Figure 1:**
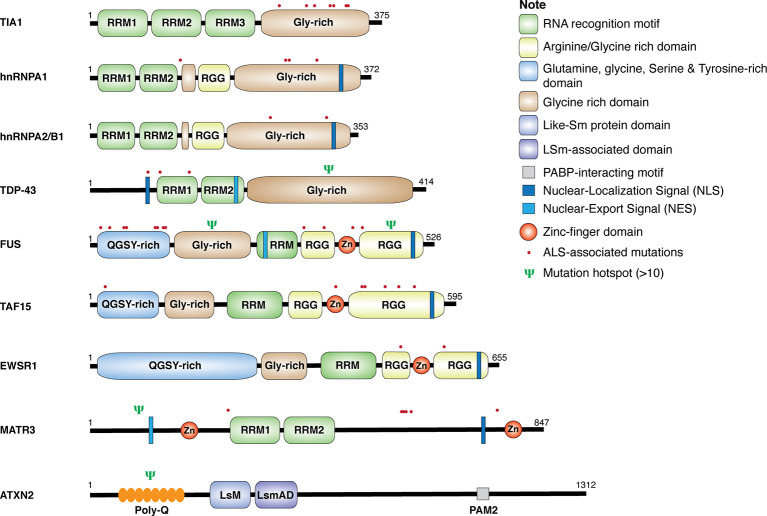
Structure and mutations in RNA-binding proteins (RBPs). Schematic diagram for amyotrophic lateral sclerosis (ALS)-associated RBPs. The location of single point mutations for each RBP is indicated as red dot, and the psi symbol denotes the location of multiple mutations associated with ALS-related motor neuron diseases.

Genetic analyses of ALS patients have identified more than 100 ALS-related gene variants, including many genes encoding RBPs, such as TDP-43, FUS, heterogeneous nuclear ribonucleoproteins (hnRNP) A1, hnRNPA2/B1, matrin 3 (MATR3), ataxin 2 (ATXN2), TATA-box binding protein–associated factor 15 (TAF15), T-cell–restricted intracellular antigen 1 (TIA-1), and Ewing sarcoma breakpoint region 1 (EWSR1; Al-Chalabi et al., 2017; Nguyen et al., 2018). As shown in Figure 1, these RBPs share some common structural domains. For example, TDP-43, hnRNPA1, hnRNPA2/B1, and TIA-1 all contain the RNA recognition motif (RRM) and the glycine (Gly)-rich prion-like domain. FUS, TAF15, and EWSR1, belonging to the thyrotroph embryonic factor (TEF) family of RBPs, share the N-terminal Gly-rich and glutamine-glycine-serine-tyrosine (QGSY)–rich prion-like domains, the RRM and zinc finger domains that facilitate RNA and DNA interactions, and the C-terminal arginine-glycine-glycine (RGG) domains that stabilize RNA and protein bindings. MATR3 harbors two RRM and two zinc-finger domains. The structure of ATXN2 is relatively unique, containing the N-terminal polyglutamine (polyQ) repeats, the like-Sm protein (LsM) and Lsm-associated domains (LsmAD) that promote RNA bindings, and the poly(A)-binding protein-interacting motif (PAMs). Except for gene mutations in *ATXN2* and *MATR3*, ALS-relevant genetic mutations in RBPs commonly occur in Gly-rich, QGSY-rich, and RGG domains (Kapeli et al., 2017). These gene mutations could lead to loss of function and/or gain of toxic function (will discuss in detail later in this review), contributing to the development of the disease.

### Posttranslational Modification

In addition to gene mutations, PTM also serves as an important mechanism for regulating protein structure and function. Aberrant PTM of RBPs is commonly observed in ALS. For example, TDP-43 is extensively posttranslationally modified, including phosphorylation, ubiquitination, acetylation, and sumoylation in both fALS and sALS (Buratti, 2018). Among them, phosphorylation of TDP-43 is the most common PTM of this protein and described as a marker of pathological ALS inclusions (Hasegawa et al., 2008). Several protein kinases have been identified to be responsible for its phosphorylation, including casein kinase (Nonaka et al., 2016), cycle 7-related protein kinase (Liachko et al., 2013), and tau tubulin kinase 1/2 (Liachko et al., 2014). Calcineurin was found to be a phosphatase that regulates TDP-43 phosphorylation (Liachko et al., 2016). Although the exact role of TDP-43 phosphorylation in disease progression remains incompletely understood, clinical data appear to support a function in neurodegeneration (Buratti, 2018).

Another key PTM associated with pathological TDP-43 is the generation of the C-terminal fragments (CTFs), which accumulate in the brains of patients with ALS or FTD and are considered a pathological feature of these diseases (Neumann et al., 2006). Several mechanisms have been proposed for their production, including proteolytic cleavage mediated by cellular proteases such as caspases and calpains, alternative splicing of the TDP-43 gene, and alternate in-frame translation (Buratti, 2018). The CTFs of TDP-43 mislocalize to the cytoplasm due to the removal of nuclear localization signal (NLS) and are prone to form aggregates as compared to the full-length TDP-43 because of the presence of Gly-rich prion-like domain. However, the relative contribution of these CTFs to disease progression remains controversial as transgenic animals expressing CTFs show only subtle motor or behavioral alterations, failing to fully recapitulate those observed in ALS or FTD patients (Berning and Walker, 2019). Methylation is another common type of PTM for RBPs. Methylation often occurs on R residues in RGG motifs of hnRNPs and TEF family of RBPs (i.e., FUS, TAF15, and EWSR1) through the action of protein arginine methyltransferases (Hofweber and Dormann, 2019). Methylation modification is a critical regulator for RBP liquid–liquid phase separation (LLPS) and dynamics of ribonucleoprotein (RNP) granules. Ribonucleoprotein granules, including stress granules (SGs) and processing bodies, are membrane-free, dense cytosolic aggregation of RBPs/RNA and common sites for mRNA storage/degradation (Buchan and Parker, 2009). Disrupted LLPS homeostasis and RNP granule dynamics have been implicated in neurodegeneration. Studies using *in vitro* systems found that R-methylation on ALS-related RBPs, such as hnRNPA2 and FUS, reduces LLPS *via* inhibiting R-aromatic interaction (Hofweber et al., 2018; Qamar et al., 2018; Ryan et al., 2018). In addition to methylation, phosphorylation has also been shown to either enhance or suppress RBP phase separation and/or RNP granule dynamics (Hofweber and Dormann, 2019).

### Disrupted Nucleocytoplasmic Trafficking

RBPs have predominant localizations within the nucleus to perform RNA processing and metabolism. However, many RBPs are abnormally aggregated in the cytoplasm in ALS. As mentioned above, the CTFs of TDP-43 are mainly found in the cytoplasmic aggregates due to the lack of the NLS. In addition, the presence of ALS-related missense mutations within NLS or PTM sites of these RBPs constitutes another mechanism responsible for their cytoplasmic accumulation (Kim and Taylor, 2017). However, gene mutations and fragmentations cannot explain all cases of the observed mislocalization of RBPs.

Emerging evidence proposes impaired nucleocytoplasmic trafficking as a key mechanism for RBP mislocalization in ALS. Although the precise mechanism remains elusive, studies suggest a role for the hexanucleotide repeat expansion mutation in chromosome 9 open reading frame 72 (*C9orf72*) gene, the most common genetic cause of ALS and FTD (DeJesus-Hernandez et al., 2011; Renton et al., 2011), in such effects. It was found that dipeptide repeats (DPRs) produced from the *C9ORF72* expansion mutant accumulate within the nuclear pore complex to disturb its integrity, leading to compromised nucleocytoplasmic transport (Freibaum et al., 2015; Jovicic et al., 2015; Zhang et al., 2015; Shi et al., 2017). Interestingly, a recent study reported that expression of C9ORF72-derived DPR poly-GA (glycine–alanine), but not poly-GR (glycine–proline) and poly-PR (proline–arginine), disturbs nucleocytoplasmic transport (Vanneste et al., 2019), suggesting a DPR-specific role in the regulation of nucleocytoplasmic trafficking. Further investigations revealed that many nucleocytoplasmic transport factors are recruited and sequestrated in the SGs upon stress or treatment with mutant proteins implicated in ALS (Zhang et al., 2018). Importantly, it was found that inhibition of SG formation attenuates the defects in nucleocytoplasmic trafficking and alleviates neurodegeneration in C9orf72-*ALS* *Drosophila* models (Zhang et al., 2018). Recent evidence has also identified a mechanism for the observed cytoplasmic mislocalization of wild-type FUS in ALS (Tyzack et al., 2019). It was found that FUS directly binds to the mRNA of splicing factor proline and glutamine rich (SFPQ). The authors proposed that translocation of SFPQ transcripts to the cytoplasm drives nuclear export of FUS (Tyzack et al., 2019).

### Aggregation and Sequestration by Abnormal RNA Foci

Protein aggregation is a common event in neurodegenerative diseases, including ALS. Several mechanisms have been recognized to contribute to protein aggregation in ALS, including self-aggregation, altered RNP granule dynamics, sequestration by aberrant RNA foci, and defects in protein quality control system (Conlon and Manley, 2017; Morriss and Cooper, 2017).

Like RNP granules, protein aggregates are formed through LLPS of weak interaction among RBPs and/or RNAs (Hofweber and Dormann, 2019). Many RBPs contain intrinsically disordered low complexity domains (LCDs) that can lead to self-aggregation, especially when abnormally mislocalized, overexpressed, or posttranslationally modified. Interestingly, it was recently reported that factors involved in nuclear import also have a role in phase transition of RBPs (Guo et al., 2018). It was shown that expression of nuclear-import receptors inhibits and reverses aberrant phase separations of RBPs, including TDP-43, FUS, hnRNPA1, hnRNPA2, TAF-15, and EWSR, to restore RBP homeostasis and rescue neurodegeneration caused by ALS-related FUS and hnRNPA1 (Guo et al., 2018). In addition, RBPs can be recruited into RNP granules through interaction with other proteins within the granules. Alternatively, abnormal RNA foci may also lead to RBP aggregations. The GGGGCC repeat RNA of *C9ORF72* can fold into G-quadruplexes and/or form stable hairpin-like secondary structures. These RNA structures sequester ALS-associated RBPs, such as TDP-43, and promote protein aggregation. *In vivo* and *in vitro* models further demonstrate that enrichment of these abnormal RNA foci can induce misprocessing of RNA transcripts that are commonly regulated by many RBPs (Conlon and Manley, 2017; Morriss and Cooper, 2017). Finally, malfunction of the protein quality control system also has a role in the accumulation of RBP aggregates. Many ALS-related mutations in genes, such as *TBK1*, *p62*, *OPTN*, *VCP*, and *UBQLN2*, are involved in autophagy and ubiquitin-proteasome degradation (Al-Chalabi et al., 2017). As a consequence of gene mutations and increased proteasomal and lysosomal load due to an excess production of abnormal protein products, the function of the protein quality control system is impaired, resulting in the buildup of RBP aggregates (Cipolat Mis et al., 2016).

## Disrupted Cellular Homeostasis Caused by ALS-Associated RBPs

**Figure 2:**
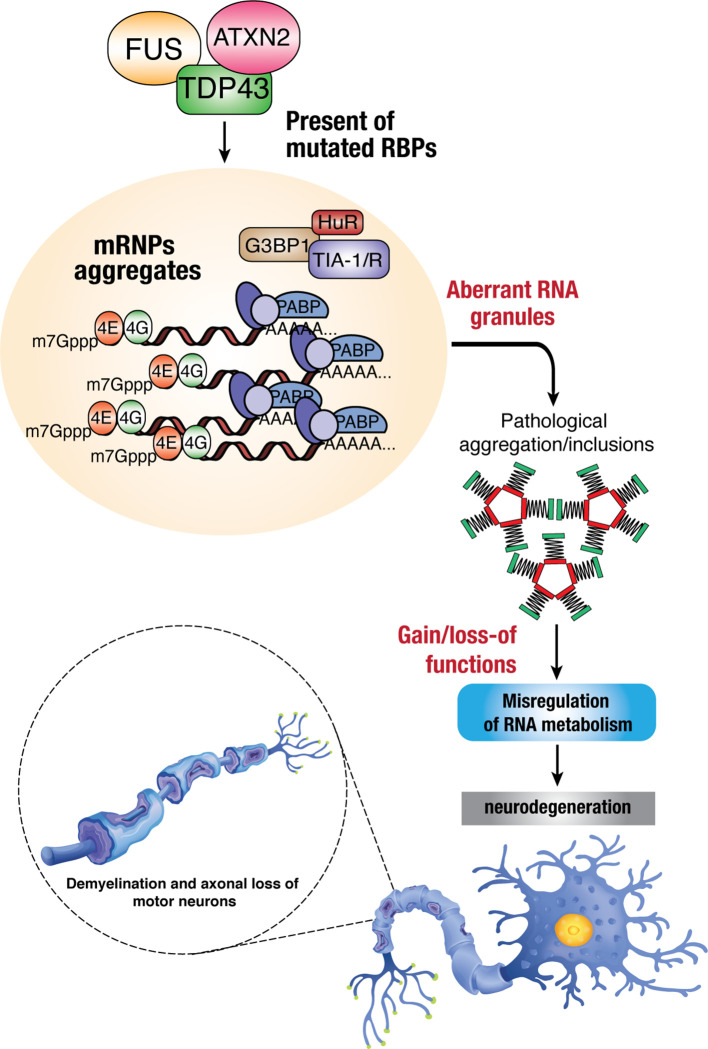
Dysregulation of RBPs in ALS. Mutations in RBPs may result in mislocalization within the cells due to disruption in nucleocytoplasmic trafficking, which can lead to the formation of toxic protein aggregates within cytoplasmic inclusions. The accumulation of these aberrant RNA granules enhances the toxicity/pathological effects in mis-regulating normal RNA metabolism and thus leading to neurodegenerative phenotypes such as demyelination, axonal loss, and death of motor neurons.

Dysregulation of RBP influences various aspects of the RNA metabolism, resulting in diverse molecular phenotypes, such as disrupted transcription and RNA splicing, abnormal RNA transport, altered mRNA stability, and protein translation. Possible mechanisms involve the loss of function and/or toxic gain of function of these RBPs through aberrant protein interactions, aggregate formation, perturbation of RNP granule dynamics, and phase transition (Figure 2). The following sections will discuss the known/speculated disease mechanisms in genes, including *TARDBP*, *FUS*, *hnRNPA1*, *hnRNPA2/B1*, *TIA1*, *TAF15*, *MATR3*, and *EWSR1*.

### TARDBP/TDP-43

TDP-43 is encoded by the *TARDBP* gene and belongs to the large family of hnRNPs (Kapeli et al., 2017). TDP-43 participates in multiple steps of RNA processing with its role in splicing being best characterized. For example, research has suggested a role for TDP-43 in cryptic splicing, which is impaired in ALS (Ling et al., 2015). The expression of TDP-43 can be autoregulated at the level of mRNA stability *via* a negative-feedback loop (Ayala et al., 2011). Under normal conditions, TDP-43 plays a vital role in maintaining the function of the central nervous system (CNS). For example, TDP-43 has been shown to regulate many mRNAs encoding proteins implicated in CNS development, survival, and synaptic transmission and neural plasticity (Polymenidou et al., 2011; Tollervey et al., 2011; Alami et al., 2014; Ling, 2018). Knockout of TDP-43 in mice is embryonic lethal, and conditional or partial loss of TDP-43 in neural cells results in progressive motor neuron degeneration and motor function impairment (Wu et al., 2012; Iguchi et al., 2013; Yang et al., 2014).

*TARDBP* was first identified as a causative gene for ALS in 2008 (Kabashi et al., 2008; Sreedharan et al., 2008; Van Deerlin et al., 2008). Since then, more than 50 mutations in *TDP-43* have been found in ALS patients, accounting for ~5% of fALS and ~1% of sALS (Taylor et al., 2016). These mutations are predominantly clustered in the Gly-rich domain, an LCD domain critical for protein aggregation and phase transition (Franzmann and Alberti, 2019). Transgenic animals expressing mutant TDP-43 were shown to develop ALS-like pathological and clinical features (Lutz, 2018). Although *TDP-43* mutations are rare, TDP-43 proteinopathies, characterized by cytoplasmic mislocalization, aggregation, and cleavage of TDP-43 accompanied by its nuclear clearance, are found in the affected regions of the CNS in up to 97% of all ALS cases, except for those fALS caused by *SOD1* or *FUS* mutations (Neumann et al., 2006; Mackenzie et al., 2010), suggesting a broader involvement for TDP-43 dysregulation in ALS. In addition to the CNS, mislocalization and aggregation of TDP-43 have also been observed in peripheral tissues, such as in the peripheral blood mononuclear cells, in both fALS (De Marco et al., 2017) and sALS (Arosio et al., 2020). The mechanisms for TDP-43 toxicity in ALS are proposed to be both a loss of function (nuclear depletion of TDP-43) and a gain of toxic function, supported by evidence that both knockout and overexpression of wild-type or mutant TDP-43 recapitulate disease phenotypes (Butti and Patten, 2018). Recent studies have established a mechanism by which aberrant RNA splicing caused by mutant TDP-43 or depletion of nuclear TDP-43 contributes to ALS through a loss of function and/or a gain of function (Deshaies et al., 2018; Fratta et al., 2018; Sivakumar et al., 2018). TDP-43 has also been implicated in the DNA damage response, and loss of nuclear TDP-43 causes defects in DNA repair associated with ALS (Mitra et al., 2019). Moreover, dysregulated or mislocalized TDP-43 has been shown to interfere with cellular translational process. For instance, it was discovered that ALS-linked cytoplasmic TDP-43 interacts with receptor for activated C kinase 1 (RACK1), a ribosomal scaffold protein, on polyribosomes, resulting in a global inhibition of protein synthesis (Russo et al., 2017). It was also shown that mutant TDP-43 (A315T) enhances direct binding and translation of several mRNAs, including *Dennd4a* and *Camta1* that are known to be involved in neurodegeneration (Neelagandan et al., 2019).

### Fused in Sarcoma/Translocated in Sarcoma

Similar to TDP-43, FUS is a DNA/RBP and has primarily nuclear localization, but mislocalizes to the cytoplasm in response to stress (Kapeli et al., 2017). FUS also plays multiple functions in RNA metabolism by binding to the target RNAs through its RRM domain (Lagier-Tourenne et al., 2012). Mutations in FUS gene were identified as a genetic driver for ALS in 2009 (Kwiatkowski et al., 2009; Vance et al., 2009). Since then, more than 70 mutations were reported in ALS patients. The majority of these mutations are localized within the NLS, QGSY-rich and RGG1 domains, leading to altered cellular localization and increased aggregation tendency. *FUS* mutations are responsible for ~5% of fALS and less than 1% of sALS cases (Taylor et al., 2016). Unlike TDP-43 pathology that can be detected in almost all ALS cases (Mackenzie et al., 2007), cytoplasmic FUS inclusion/aggregation is uncommon and observed only in *FUS*-related fALS (Vance et al., 2009) and a very small subset of sALS (Deng et al., 2010). Thus, the contribution of FUS to ALS did not receive as much attention as that of TDP-43. However, a recent study found that cytoplasmic FUS in a diffused form is widely present in ALS, suggesting that it may have a broader role in ALS than previously recognized (Tyzack et al., 2019). This speculation warrants future investigations.

Unlike TDP-43, the function of FUS does not appear to be essential for embryonic development. Global or motor neuron–specific knockout of *FUS* gene in mice does not cause motor deficits (Sharma et al., 2016), whereas transgenic mice expressing wild-type or mutant *FUS* were shown to develop severe motor impairments and exhibit progressive neurological symptoms, suggesting a mechanism of toxic gain of function rather than loss of function in *FUS*-linked ALS pathogenesis (Qiu et al., 2014; Scekic-Zahirovic et al., 2017; López-Erauskin et al., 2018). Although the exact mechanisms of FUS toxicity are still unclear, recent studies began to unravel the involvement of defective RNA binding and splicing, disrupted protein translation, and impaired DNA damage response. ALS–associated mutant FUS has been shown to have altered RNA binding profiles (Hoell et al., 2011), related to the alteration of its subcellular localization (Dormann et al., 2010; Deshpande et al., 2019). Mutant FUS can also cause splicing defects in genes involved in dendritic growth and synaptic functions (Qiu et al., 2014). In addition, ALS-related mutant FUS was found to suppress protein translation at both global and local (axon regions) levels and disrupt the nonsense-mediated decay, both of which are associated with motor neuron pathogenesis seen in human (Kamelgarn et al., 2018; López-Erauskin et al., 2018). Moreover, defects in DNA damage repair were also proposed to be a mechanism underlying *FUS*-induced ALS pathogenesis. It was reported that DNA damage repair is impaired in human induced pluripotent stem cell (iPSC)—derived motor neurons that carry *FUS* mutations, leading to the formation of cytoplasmic aggregates and neurodegeneration (Naumann et al., 2018; Wang et al., 2018). Finally, a recent study revealed that activation of antiviral immune response is sufficient to induce the formation and persistency of cytoplasmic FUS-containing aggregates, contributing to onset and progression of FUS proteinopathy (Shelkovnikova et al., 2019).

### hnRNPA1 and hnRNPA2/B1

hnRNPA1 and hnRNPA2/B1 are two essential RBPs in the family of hnRNPs. Like other RBPs, hnRNPA1 and hnRNPA2/B1 are typically nuclear localized but accumulate in the cytoplasm under stress conditions (Kapeli et al., 2017). hnRNPA1 and hnRNPA2/B1 share similar functions in the regulation of mRNA maturation, splicing, translation, and stability, but they play a differential role in transcriptional regulation (Kapeli et al., 2017). While knockout of *hnRNPA1* is embryonic lethal, and heterozygous mice display defects in muscle development (Liu et al., 2017), knockdown of hnRNPA2/B1 in mice globally disrupts alternative splicing and causes impaired cognitive function (Berson et al., 2012). Genetic analysis identified several ALS-related mutations in both *hnRNPA1* and *hnRNPA2/B1* (Kim et al., 2013; Couthouis et al., 2014; Liu et al., 2016; Naruse et al., 2018); however, the incidence is low (<1%; Taylor et al., 2016). Although the consequence of these mutations is largely unclear, some evidence suggests that mutations within the LCD could lead to increased cytoplasmic accumulation of hnRNPA1 through misfolding and fibrilization that stabilize the proteins (Kim et al., 2013; Gilpin et al., 2015; Molliex et al., 2015; Liu et al., 2016). It was also reported that altered *hnRNPA1* splicing induced by mutant or nuclear depletion of TDP-43 results in the production of an aggregation-prone mutant form of hnRNPA1, contributing to disease progression (Deshaies et al., 2018; Sivakumar et al., 2018). Furthermore, mutations of genes are expected to cause a disruption of protein synthesis as both hnRNPA1 and hnRNPA2/B1 have been implicated in protein translation (Kosturko et al., 2006; Cammas et al., 2007). Finally, iPSC-derived motor neurons expressing ALS-linked hnRNPA2/B1 mutant were shown to have a higher level of cell death and increased stress responses (Martinez et al., 2016).

### Others RBPs, Including TIA-1, MATR3, ATXN2, TAF15, and EWSR1

TIA-1 is a critical component of SGs and has multiple functions in RNA metabolism, including mRNA splicing, translational repression, and mRNA silencing. TIA-1 is mainly localized to the nucleus; however, under cellular stress, it translocates to the cytoplasm, where it nucleates SGs and suppresses mRNA translation (Rayman and Kandel, 2017). A number of ALS-related missense mutations in *TIA-1* gene have been identified, all of which manifest in the Gly-rich domain (Mackenzie et al., 2017). Mutations of *TIA-1* gene were shown to alter LLPS and impair SG disassembly *in vitro* (Mackenzie et al., 2017). Nonetheless, because of their rare occurrence in patients, the causality between mutations in *TIA-1* and the pathogenesis of ALS is still debated (Baradaran-Heravi et al., 2018; van der Spek et al., 2018).

MATR3 is a DNA/RBP that interacts with TDP-43. Similar to other RBPs, MATR3 is involved in different steps of RNA processing, including gene transcription, alternative splicing, mRNA export, and stability (Malik et al., 2018). *MATR3* mutations were first reported to be linked to ALS in 2014 (Johnson et al., 2014). Like *TIA-1*, mutations in *MATR3* are rare (<1%) among ALS patients (Taylor et al., 2016). Studies from two recent articles showed that mice expressing mutant *MATR3* (*S85C* or *F115C*) develop motor dysfunction, accompanied by decreased numbers of motor neurons and activation of microglia and astrocytes in the spinal cord (Moloney et al., 2018; Zhang et al., 2019). Mechanistically, it was found that ALS-linked mutations of this gene decrease mRNA nuclear export and inhibit SG formation (Boehringer et al., 2017). S85C mutation of MATR3 was also shown to disrupt the normal function of MATR3 in mediating phase separation and formation of intranuclear droplets (Gallego-Iradi et al., 2019). Contrary to other ALS-related RBPs, wild-type or mutant MATR3 is primarily localized to the nucleus even under stress or in ALS postmortem tissues (Johnson et al., 2014; Gallego-Iradi et al., 2015), and the accumulation of MATR3 in the nucleus as opposed to its location in the cytoplasm mediates neurotoxicity (Malik et al., 2018).

ATXN2 is an RBP belonging to the like-Sm (LSm) family and participates in the regulation of RNA metabolism. The *ATXN2* gene normally has ~22–23 glutamine (CAG) repeats. Intermediate-size polyQ expansions (24–34 repeats) have been discovered to be significantly associated with the risk of developing ALS (Elden et al., 2010; Sproviero et al., 2017). ATXN2 plays a critical role in regulating TDP-43 and FUS toxicity through direct protein–protein interaction, and reduction of ATXN2 levels has been shown to inhibit TDP-43–mediated neurotoxicity (Elden et al., 2010; Farg et al., 2013; Becker et al., 2017). Both *in vitro* and knock-in mouse studies demonstrated that polyQ expansion mutants increase the insolubility of ATXN2 and its interacting protein PABPC1, causing neurodegeneration (Damrath et al., 2012).

Mutant forms of both *TAF15* and *EWSR1* have also been identified in ALS patients (Couthouis et al., 2011, 2012; Ticozzi et al., 2011). Similar to other RBPs, TAF15 and EWSR1 normally reside in the nucleus, but translocate to the cytoplasm upon stress (Neumann et al., 2011; Marko et al., 2012). Expression of disease-related variants of TAF15 and EWSR1 in primary neurons from mouse spinal cord has been reported to cause the formation of cytoplasmic foci. Cytoplasmic TAF15 and EWSR1 aggregates were also detected in some spinal cord neurons of patients with sALS (Couthouis et al., 2011, 2012). Overexpression of wild-type and mutant *TAF15* and *EWSR*1 has been shown to enhance protein aggregation and shorten life span in *Drosophila* (Couthouis et al., 2011, 2012).

## Development of Novel Therapeutics by Targeting RBPs

Early studies on ALS therapy have been mostly focused on SOD1. However, SOD1 mutations account for approximately only 20% of fALS and approximately 2% to 3% of all ALS cases (Taylor et al., 2016). Given the recognized importance of RBPs in ALS, RBPs have emerged as critical therapeutic targets for the treatment of ALS.

### Antisense Oligonucleotide

Antisense oligonucleotides (ASOs) are synthetic single-stranded oligonucleotides that can specifically bind to and accelerate the degradation of the target mRNA *via* the nuclear endonuclease RNase H. ASO-based gene silencing has been previously tested on mutant *SOD1* (m*SOD1*), and the results are promising (Smith et al., 2006; Miller et al., 2013; McCampbell et al., 2018). It was found that direct delivery of m*SOD1*-targeted ASOs to the CNS effectively reduces the levels of mSOD1 and significantly delays disease progression and prolongs survival in SOD^G93A^ animal models (Smith et al., 2006; McCampbell et al., 2018) and mSOD1-linked ALS patients (Miller et al., 2013). Recently, this technique has been utilized to target ATXN2, a key regulator of TDP-43 neurotoxicity, for degradation (Becker et al., 2017). It was shown that administration of *ATXN2*-targeted ASOs to the CNS of a mouse model with TDP-43 proteinopathy attenuates TDP-43 pathology and improves motor function (Becker et al., 2017). Moreover, ASO-based strategies have also been successfully used to silence *GGGGCC* repeat expansion of *C9orf72*, thereby inhibiting RBPs sequestration by abnormal RNA foci (Donnelly et al., 2013; Sareen et al., 2013).

### Small Molecules

Small molecules serve as another strategy for ALS treatment by modulating the function and abundance of RBPs. For instance, small molecules that specifically bind to the RRMs (RRM1 and RRM2) of TDP-43 preventing pathogenic interaction of TDP-43 and RNAs led to motor function improvement *in vivo* (*Drosophila*, François-Moutal et al., 2019). In addition, inhibition of tankyrase, a poly(ADP-ribose) polymerase, through small molecules was shown to reduce the accumulation of cytoplasmic TDP-43, FUS, or HNRNPA2/B1 aggregates and associated pathologies (McGurk et al., 2018; Fang et al., 2019; Marrone et al., 2019). Various small molecule activators of autophagy, a major cellular pathway for disposing of misfolded protein aggregates and damaged organelles, have also been tested in mutant *TDP-43* transgenic mice and in mutant *FUS* *Drosophila* models (Wang et al., 2012; Barmada et al., 2014; Marrone et al., 2019). It was shown that application of these autophagy inducers increases the clearance of protein aggregates and improves motor functions and pathologies. Finally, studies using chemical inhibitors to lessen nuclear export of TDP-43 demonstrated a neuroprotective effect (Haines et al., 2015).

### Chaperones

Molecular chaperones, such as heat shock proteins (HSPs), function to assist proper protein folding and prevent misfolded proteins from aggregation. In addition, chaperones can also guide terminally misfolded proteins to the proteolytic system for degradation (Xiao et al., 2010). In the study of ALS treatment, modified yeast HSP104 was discovered to rescue TDP-43 and FUS proteotoxicity by promoting aggregate dissolution (Jackrel et al., 2014). Moreover, HSPB8, a small HSP, was reported to promote autophagic clearance of misfolded mutant TDP-43 and SOD1, as well as DPRs of C9orf72 (Crippa et al., 2016). Notably, a randomized phase II clinical trial is undertaken to examine the therapeutic value of colchicine, which induces the expression of HSPB8 and several autophagy modulators, in ALS (Mandrioli et al., 2019).

### Antibodies

Antibody-based therapies have been widely explored for the treatment of neurodegenerative diseases, including ALS. There was a recent study showing beneficial effects of antibodies targeting TDP-43 in ALS (Pozzi et al., 2019). In this study, a single-chain antibody against the RRM1 of TDP-43, a domain involved in protein aggregation and interaction with p65 nuclear factor κB, was generated and delivered into the CNS of TDP-43 mutant transgenic mice through a viral vector. It was found that antibody treatment in these mice significantly reduces neuroinflammation, cognitive impairment, and motor defects (Pozzi et al., 2019).

## Conclusion

The contribution of dysfunctional RBPs in both fALS and sALS has been greatly appreciated over the last decade since the recognition of *TDP-43* as an ALS causal gene and TDP-43 proteinopathy as a hallmark for ALS. The ALS-implicated RBPs share structural and functional similarity. Dysregulated or mutant RBPs have been shown to cause disease phenotype through common protein pathologies, including increased aggregation tendency, cytoplasmic mislocalization, and irregular LLPS and SG dynamics. However, current evidence also points to a distinct function and unique mechanism for individual RBPs in the pathogenesis of ALS. A better understanding of the molecular mechanisms underlying the pathological role of these RBPs in ALS development will lead to a novel avenue for therapeutic intervention for this devastating disease.

## Author Contributions

All authors contributed to the writing of this article.

## Conflict of Interest

The authors declare that the research was conducted in the absence of any commercial or financial relationships that could be construed as a potential conflict of interest.
